# Breeding Drought-Tolerant Pearl Millet Using Conventional and Genomic Approaches: Achievements and Prospects

**DOI:** 10.3389/fpls.2022.781524

**Published:** 2022-04-07

**Authors:** Rakesh K. Srivastava, O. P. Yadav, Sivasakthi Kaliamoorthy, S. K. Gupta, Desalegn D. Serba, Sunita Choudhary, Mahalingam Govindaraj, Jana Kholová, Tharanya Murugesan, C. Tara Satyavathi, Murali Krishna Gumma, Ram B. Singh, Srikanth Bollam, Rajeev Gupta, Rajeev K. Varshney

**Affiliations:** ^1^International Crops Research Institute for the Semi-Arid Tropics (ICRISAT), Patancheru, India; ^2^Indian Council of Agricultural Research-Central Arid Zone Research Institute, Jodhpur, India; ^3^United States Department of Agriculture-Agriculture Research Service (ARS), U.S. Arid Land Agricultural Research Center, Maricopa, AZ, United States; ^4^Indian Council of Agricultural Research – All India Coordinated Research Project on Pearl Millet, Jodhpur, India; ^5^United States Department of Agriculture-Agriculture Research Service (ARS), Edward T. Schafer Agricultural Research Center, Fargo, ND, United States; ^6^State Agricultural Biotechnology Centre, Centre for Crop & Food Innovation, Food Futures Institute, Murdoch University, Murdoch, WA, Australia

**Keywords:** drought stress, drought mechanism, drought tolerance, genetic resources, genomic resources

## Abstract

Pearl millet [*Pennisetum glaucum* (L.) R. Br.] is a C_4_ crop cultivated for its grain and stover in crop-livestock-based rain-fed farming systems of tropics and subtropics in the Indian subcontinent and sub-Saharan Africa. The intensity of drought is predicted to further exacerbate because of looming climate change, necessitating greater focus on pearl millet breeding for drought tolerance. The nature of drought in different target populations of pearl millet-growing environments (TPEs) is highly variable in its timing, intensity, and duration. Pearl millet response to drought in various growth stages has been studied comprehensively. Dissection of drought tolerance physiology and phenology has helped in understanding the yield formation process under drought conditions. The overall understanding of TPEs and differential sensitivity of various growth stages to water stress helped to identify target traits for manipulation through breeding for drought tolerance. Recent advancement in high-throughput phenotyping platforms has made it more realistic to screen large populations/germplasm for drought-adaptive traits. The role of adapted germplasm has been emphasized for drought breeding, as the measured performance under drought stress is largely an outcome of adaptation to stress environments. Hybridization of adapted landraces with selected elite genetic material has been stated to amalgamate adaptation and productivity. Substantial progress has been made in the development of genomic resources that have been used to explore genetic diversity, linkage mapping (QTLs), marker-trait association (MTA), and genomic selection (GS) in pearl millet. High-throughput genotyping (HTPG) platforms are now available at a low cost, offering enormous opportunities to apply markers assisted selection (MAS) in conventional breeding programs targeting drought tolerance. Next-generation sequencing (NGS) technology, micro-environmental modeling, and pearl millet whole genome re-sequence information covering circa 1,000 wild and cultivated accessions have helped to greater understand germplasm, genomes, candidate genes, and markers. Their application in molecular breeding would lead to the development of high-yielding and drought-tolerant pearl millet cultivars. This review examines how the strategic use of genetic resources, modern genomics, molecular biology, and shuttle breeding can further enhance the development and delivery of drought-tolerant cultivars.

## Introduction

Pearl millet [*Pennisetum glaucum* (L.) R. Br. *syn. Cenchrus americanus* L.] has been widely grown in Sahelian West Africa and the Indian subcontinent since time immemorial. Archeological evidence (Manning et al., 2011) as well as modern genome sequence analysis (Burgarella et al., 2018) indicated that pearl millet originated and was domesticated in West Africa about 4,000 to 5,000 years ago. Pearl millet is grown in an agriculturally harsh environment where other staple crops are likely to fail in producing a reasonable grain yield. Pearl millet is a momentous food crop around the globe after maize (*Zea mays*), wheat (*Triticum aestivum*), rice (*Oryza sativa*), sorghum (*Sorghum bicolar*), and barley (*Hordeum vulgare*). It is an important eco-friendly field crop in conventional farming, which is good for human nutrition and health, and the planet. Pearl millet may counter several aversive impacts of looming climate change on food grain production better than other staple cereals and plays a significant role in food and nutritional security. To enhance genetic gains in terms of grain yield, nutritional values, climate resilience, and multiple disease resistance, constant efforts will be needed to develop more potential genomic resources for the pearl millet species (Srivastava et al., 2020a).

Usually, pearl millet is grown not only for nutritious grains but also for grazing, silage, hay, green chop, poultry feed, dry stover, and bio-energy. Pearl millet grains have been consumed as a staple food for thousands of years to make a diversity of food stuffs, and continue to be used as a staple grain by millions of people in African and Asian countries. Pearl millet is a perfect alternative for low-input sustainable agricultural systems. Regardless of its lower productivity owing to various environmental stresses (terminal drought and flowering-stage heat) and biotic constraints (diseases like downy mildew, blast, rust, ergot, and smut), pearl millet produces nutritive grains, which are a resource for addressing malnutrition among the poor in marginal areas of the world (Rai et al., 2013).

Pearl millet has a very efficient energy production system (C_4_ photosynthesis) for rapid production of biomass that partly has enabled it to adapt to suboptimal arid and semi-arid environments around the world (Wang et al., 2012). The high degree of tolerance to high temperature during the early and reproductive stages also makes it a promising genetic resource for isolation of candidate genes governing adaptation to suboptimal growing conditions (Gupta et al., 2015). High tillering capacity and cross-pollination that produces heterogeneous plants contributed in its resilience to hot and dry environments (Kumar and Andrews, 1993). Pearl millet tillers freely and compensates for any stand irregularity due to early drought, and it produces several heads per plant depending on moisture availability. Today, pearl millet is grown in over 30 million hectares of land and supports more than 100 million people as a staple food, and different forms are found in the Sahel extending from Senegal to Sudan (Brunken, 1977).

Limited soil moisture content is one of the key environmental stresses affecting grain yield and nutritional quality. The looming climate change is expected to make environmental stresses even more aggravated (Kholová et al., 2010a). Hence, sustainable food and nutritional security will rely on breeding cultivars with higher adaptation traits in water-limited environments. Several efforts have been attempted to enhance water use efficiency in dry lands, and have resulted in significant improvements in adaptation to drought stress in pearl millet cultivars (Yadav et al., 2002; 2004; 2011; Serraj et al., 2005; Bidinger et al., 2007; Vadez et al., 2012; Varshney et al., 2017). Genetic improvement in several key traits has lagged behind other staple cereals, but there is good potential to enhance pearl millet resilience to environmental stresses. Availability of the pearl millet whole-genome sequence (Varshney et al., 2017) paves the way to exploit its wide genetic variability to develop cultivars and hybrids well suited to present and environmental stresses (Debieu et al., 2017). This review attempts to assess the progress made in mapping of drought environments in the pearl millet growing ecology, understand the response of pearl millet to various types of drought, comprehend the mechanism of pearl millet drought adaptation, scrutinize procedures and advanced techniques to dissect drought tolerance, and assess the role of genomic resources in breeding for drought tolerance. We contend that understanding these aspects would greatly advance the development of drought-tolerant pearl millet cultivars at a faster rate.

## Response of Pearl Millet to Drought

The future climate will likely see increased drought frequency, varied precipitation patterns, and unpredictability of monsoon rains, which can cause drought in different stages of plant growth in different environments. It will make the breeding process even more difficult if studies on traits that drive adaptation are not growth stage-specific and well-characterized for each stage. Accordingly, cost-effective phenotyping of identified traits can be meticulously integrated with pearl millet breeding programs focusing on environment-specific strategy.

### Seedling Stage

Seedling stage drought is prominent in crop production regions receiving rainfall lower than 400 mm per annum such as Thar desert (Western Rajasthan, India) where seedling establishment itself is a major challenge (Howarth et al., 1996). Most breeding programs fail to deliver products for drier regions mainly because of narrow genetic variations for seedling establishment and vast variations in microclimate (day and night temperature variations and humidity) and water-holding capacity of the soil; apart from rainfall, which requires proper quantification. Early season soil moisture stress results in seedling mortality, leading to poor crop establishment and reduced yield in pearl millet (Carberry et al., 1985; Yadav et al., 2012a). Genetic variation has been reported for root and shoot length and its ratio, and leaf formation and secondary root development in early season moisture stress (Gregory, 1983; Govindaraj et al., 2010; Shivhare and Lata, 2019; Mukesh Sankar et al., 2021). Agronomic management plays very little role in reducing seedling mortality, but it was reported that early seedling vigor along with larger seed size could enhance crop stand establishment contributing to drought adaptation (Howarth et al., 1996).

### Vegetative Stage

Drought stress in the vegetative stage is of frequent occurrence and seriously affects pearl millet growth (Kholová et al., 2010a; Medina et al., 2017; Shivhare and Lata, 2017), tillering ability (van Oosterom et al., 2003, 2006), and flowering (Bidinger et al., 1987a; Mahalakshmi et al., 1987), which are important components of crop productivity. The high-tillering nature of pearl millet compensates for loss of panicles in the main shoot under intermittent drought provided that water is available at a later season. This feature helps pearl millet to adapt to drought as it takes advantage of late rain to produce additional productive tillers (Siband, 1983; Mahalakshmi et al., 1987). Also, early flowering is an important attribute of pearl millet for drought escape, however, constraints in predicting rainfall events in drier regions limit the breeding process for early flowering traits (Vadez et al., 2012).

### Reproductive Stage

Soil moisture stress in the flowering and post-flowering stages are common because of early cessation of rainfall in the growing season. In fact, collectively, terminal drought stress accounts for a huge impact on pearl millet grain and stover yields (Mahalakshmi et al., 1987; Winkel et al., 1997; Bidinger and Hash, 2004; Kholová and Vadez, 2013). Grain yield penalty is maximum when drought strikes during the flowering and grain filling stages of the crop (Lahiri and Kumar, 1966; Mahalakshmi et al., 1987) owing to decrease in the number of fertile florets per panicle and grain size in pearl millet (Bidinger et al., 1987a; Fussell et al., 1991). Reduction in grain size is mainly due to shortening of the period for grain filling rather than reduction in the rate of grain growth (Henson et al., 1984; Bieler et al., 1993), as pearl millet also has an exceptional capacity to compensate for reduction in the supply of assimilates to the grains by mobilizing stored soluble sugars in stems and leaves (Fussell et al., 1980). The contribution of stored assimilates to grain growth during drought stress has, however, not been quantified (Yadav et al., 2012c). Connection between grain development and transfer of assimilates from the leaves has been reported as the central adaptation mechanism in pearl millet to terminal drought stress (Winkel and Do, 1992). However, more recent evidence shows that pearl millet adaptation to terminal drought stress depends on soil moisture availability during the grain-filling stage. Therefore, water-saving mechanisms like transpiration efficiency, restricted transpiration during high atmospheric demand, and leaf expansion at a lower threshold of soil moisture can sustain photosynthesis for continued carbon supply to the grains during the critical period (Vadez et al., 2013; Choudhary et al., 2019).

It has been convincingly shown that the effects of drought are influenced by the developmental stage of the crop in which drought stress occurs, which is environment-specific. Therefore, the development of drought-tolerant products requires proper quantification of the environment and then fit-for-purpose product development through innovative breeding.

## Physiological Basis of Drought Adaptation

Grain yield is a complex process influenced by several component traits at the bottom of plant structural organization and is the consequence of interaction among the environment, management, and genotype. Below are several physiologically important traits reported to play a critical role in drought adaptation of pearl millet.

### Soil Water Conservation Mechanism

**FIGURE 1:**
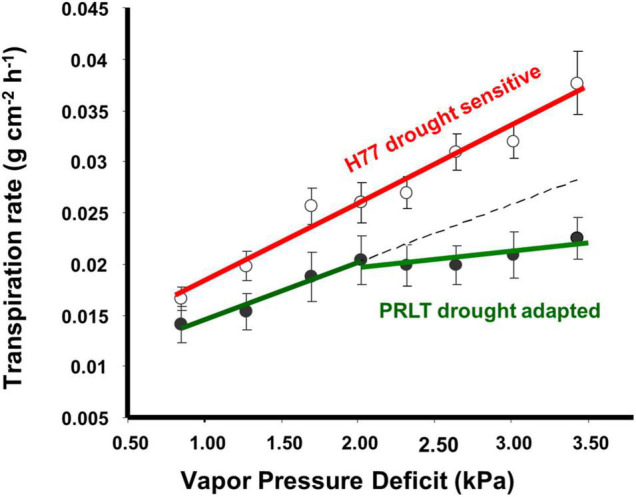
Pearl millet genotypes contrasting for drought adaptation were shown to differ in their transpiration response to increase in vapor pressure deficit (VPD, adopted from Kholová et al., 2010b).

**FIGURE 2:**
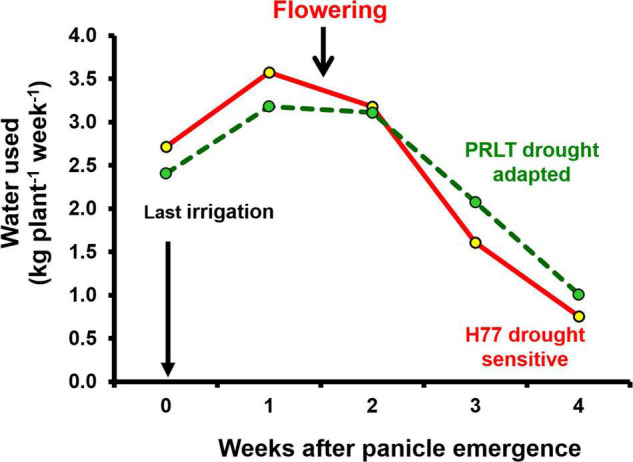
Water extraction pattern in H77 and PRLT before and after flowering under water deficit conditions (adopted from Vadez et al., 2013).

Availability of an adequate amount of water during critical growth stages determines the productivity of crop plants. Terminal drought-adapted genotypes have built-in plant traits such as lower transpiration rate (Figure 1) and lower water extraction pattern (Figure 2) that promote conservation of soil water in early growth stages to allow for more water to be available for effective grain filling during terminal drought (Vadez et al., 2013). There is a significant relationship between transpiration rate and soil moisture content observed in pearl millet (Kholová et al., 2010a).

Water extraction pattern in critical crop growth stages: the pattern of water extraction in pearl millet has been explored using the lysimetric facility simulating field conditions (Vadez et al., 2013). When irrigation was withheld in the late vegetative stage, the drought-adapted genotype PRLT-2/89-33 showed less water extraction before flowering, and conserved soil moisture was hypothesized to support grain filling, whereas the drought-sensitive genotype H77/833-2 extracted more water before flowering, and depleted soil moisture could not support grain filling (Tharanya et al., 2017).

### Limited Transpiration Rate at High Atmospheric Evaporative Demand

**FIGURE 3:**
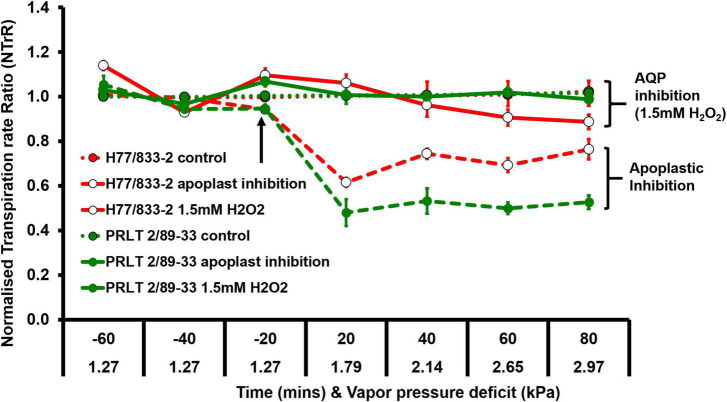
Genotypic difference in transpiration response of H77/833-2 (high Tr, open circle) and PRLT-2/89-33 (low Tr, closed circle) to AQP and apoplastic inhibition (adopted from Tharanya et al., 2018).

Plants do suffer from a type of stress called atmospheric drought when the evaporative demand (vapor pressure deficit, VPD) of the environment is high especially under midday conditions irrespective of soil moisture status. During high VPD conditions, transpirational water loss is higher, and photosynthetic efficiency is low; in other words, plants spend a lot of water molecules in transpirational cooling with very minimum CO2 fixation (Figure 3). Limited TR under increasing atmospheric VPD was identified as one of the water-saving mechanisms and genotypic variations were reported in pearl millet (Kholová et al., 2010b; Medina et al., 2017). The limited TR trait in pearl millet operates by hydraulic regulation and partial closure of stomata contributing to water conservation. Drought-adapted genotypes have lower transpiration rates at high atmospheric evaporative demand than terminal drought-sensitive lines (Kholová et al., 2010a,b; Medina et al., 2017; Tharanya et al., 2018; Choudhary et al., 2019). The genotypes contrasting for transpiration rate under elevated VPD condition was found to differ in their dependence on water channeling pathway mediated through aquaporins which play an important role in hydraulic regulation (Tharanya et al., 2018).

### Canopy Development and Expansion Threshold Under Soil Moisture Stress

Canopy development traits are closely associated with crop water use (Tharanya et al., 2018) and are a combination of vigor and size (Vadez et al., 2015). Vadez et al. (2015) looked at the difference in canopy development of pearl millet hybrids bred for different agro-ecological zones of India and hypothesized that these hybrids may have a difference in canopy development resulting in variation in leaf area. F_1_ hybrids developed for the A_1_ dry zone produced smaller leaf areas and lower biomass than materials bred for the relatively wetter A and B zones. These differences in canopy development were also reported to result in water conservation under water deficit conditions (Vadez et al., 2015; Choudhary et al., 2019), which had a genetic basis (Yadav et al., 2005; Kholová et al., 2012; Tharanya et al., 2018).

### Leaf Abscisic Acid Deposition Under Water Stress

Transpiration is known to be regulated by a combination of hydraulic signals and biochemical mediators like abscisic acid (ABA) (Comstock, 2002). It is postulated that although higher production of ABA may decrease stomatal conductance, it can increase root hydraulic conductivity (Thompson et al., 2007). Grantz (1990) reported that leaf stomatal closure at high evaporative demand from the atmosphere could be mediated by ABA. Under well-watered conditions, the drought-adapted pearl millet genotype PRLT-2/89-33 was found to have high leaf ABA during the vegetative stage.

## Modeling Drought in the Context of G × E and Identification of Environment-Specific Morphophysiological Traits

The interaction of genotypes with the environment restrains genetic gain and insights into drought adaptation. Therefore, it is important to characterize the environment in which a crop is grown. A few decades back, the All-India Coordinated Pearl Millet Improvement Project (AICPMIP) defined the pearl millet growing area of the country into three separate zones, A1 (the arid zone of NW India), A (the higher rainfall areas of north and central India), and B (semi-arid peninsular India). Each of these zones covers areas with high variation in agro-climatic conditions that have, in these decades, also changed dramatically. Recognizing this current variation in existing zones redefining pearl millet-growing area and identifying crop production constraints (drought stress patterns) will enable breeders to understand the need for breed-specific cultivars for each target agro-ecology using a suitable breeding strategy. Crop modeling is also highly useful for designing ideal plant ideotypes based on evaluation of past genetic improvement for the selected environment. The efficiency of crop improvement for constantly changing environments can be improved if the physiological and morphological traits associated with drought adaptation are identified and integrated into breeding programs. Singh et al. (2017) used a modified CSM-CERES-Pearl millet model to study the effect of altered traits determining the maturity of the crop, its yield and adaptation to heat and drought prevailing in semi-arid regions of India and Africa. It was found that decreasing crop maturity duration had a negative impact on yield, and that increasing the maturity duration benefited yield in few locations under current and future predicted climatic conditions. In addition, increasing radiation use efficiency (RUE) resulted in higher yields under climate change conditions. Also, improving the length and depth of roots is recommended as an important mechanism for drought adaptation and achievement of better yield (Vadez et al., 2012). The interaction of genotypes with the environment usually results in hampering of the progress of crop breeding programs. Therefore, it is essential to understand the underlying physiological process behind the interaction (Basford and Cooper, 1998). It was reported that pearl millet hybrids suitable for the arid zone must have a smaller canopy than hybrids bred for non-arid regions, and it was related to water use (Vadez et al., 2015). Differences in pearl millet genotypic transpiration responses and growth showed that breeding for various agroecological zones reflected in breeding of specific plant strategies associated plant water use related traits (Medina et al., 2017). van Oosterom et al. (2003) explained the local landraces’ adaptation to drought environments by analyzing the yield components of pearl millet and concluded that landraces benefited from limited water availability as they produced small-sized main shoots panicles. Tharanya et al. (2018) showed that in environments with unlimited water access, pearl millet genotypes with high tillering and biomass accumulation led to increase in water use and eventually higher yield. On the contrary, low plant vigor with less tiller and size along with stay-green trait favored yield in drier regions. In this regard, it is evident that the effect of the interaction of genotype with the environment determines the success of drought adaptation. It is also demonstrated that traits that favored yield in a specific environment might bring a production penalty in another (Yadav and Weltzien, 2000). Therefore, it is essential to characterize the environment for a better understanding of genotype-environment interactions. A detailed characterization of pearl millet growing area through a modeling approach and identification of crop production constraints (drought stress patterns) will enable breeders to understand each target agro-ecology for breeding specific cultivars.

## Phenotyping Pipeline to Support Drought Breeding Programs

Drought is a complex trait, and breeding for sustainable drought products requires knowledge of the type of drought stress to target the environment and systematic screening method/tool relevant to the stress. Screening methods should be precise and reproducible to accommodate the different number of breeding lines for selection in order to integrate in the breeding pipeline. A combination of screening methods can be integrated in different stages of the breeding pipeline for drought product development.

### Sensor-Based Methods for Large Scale Phenotyping in Early Breeding Stage

Integration of sensor-based smart digital technologies like HTP phenotyping platform and UAV based field phenotyping methods can accelerate the breeding process in early stages (F4, F5) of breeding schema for simple component traits of adaptation like early vigor, canopy index, plant height, and tiller number can be useful for discarding non-desirable phenotypes not only for agronomic traits but also for adaptation traits. One such high-throughput and automated phenotyping platform, “LeasyScan,” was established at ICRISAT^1^ to screen various plant materials in a non-destructive manner during the vegetative stage using an optical system (PlantEye^®^^2^). This can be used for precise measurements of plant canopy traits such as digital biomass, 3D-leaf area, plant height, leaf area index, leaf angle, leaf inclination, and light penetration depth (Vadez et al., 2015; Sivasakthi et al., 2018, 2019; Tharanya et al., 2018). This phenotyping platform is also capable of detecting plant diurnal variations in water use parameters using 1,500 analytical scales (Kar et al., 2020). A pearl millet fine-mapping population (*n* = 162), segregated for QTLs for drought adaptation (Tharanya et al., 2018), was used for phenotyping canopy development [3D-leaf area (3DLA), projected leaf area (PLA), plant height, leaf area index, canopy structure] and conductance (evapotranspiration, transpiration, and transpiration rate)-related traits (Tharanya et al., 2018). This study revealed a large genotypic variation for most of the traits which were not clear in the pot culture experiment. In addition, the principal component analysis revealed an effect of canopy structure on early water use (Lysimetric study) and grain yield (field) obtained under moderate or no water stress (Tharanya et al., 2018). There are no reports on UAV-based characterization in pearl millet under drought conditions.

### Precise Characterization of Selected Lines for Water Use Traits

**FIGURE 4:**
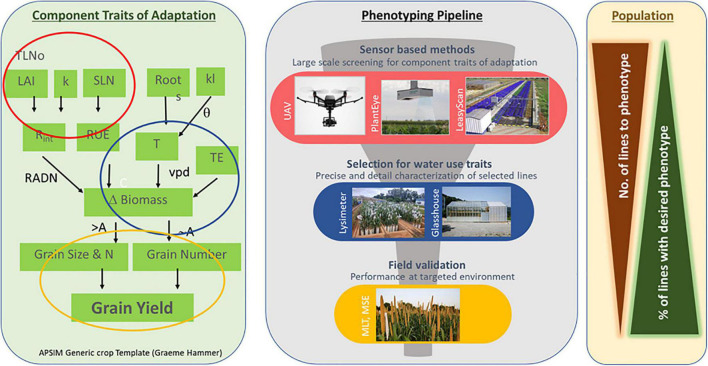
Phenotyping principle supporting drought breeding programs.LAI, leaf area index; TL No, tiller number; SLN, specific leaf nitrogen; Rint, radiation intercept; RUE, radiation use efficiency; RADN, radiation; kl, crop rooting parameters; T, transpiration; TE, transpiration efficiency; –VPD, vapor pressure deficit; Grain N, grain s; A, assimilation; UAV, unmanned aerial vehicle; MLT, multilocation testing; MSE, multisite evaluation.

Selected lines from large scale screening can be taken for detail and precise characterization for drought type pertaining to targeted ecologies (Figure 4). This can be done by artificial imposition of water stress and water uptake measurements under rainout shelter or glasshouse conditions. Screening using the lysimeter platform^3^ is a gravimetric system of transpiration measurement providing individual soil profile to each plant, and water use could be measured throughout the crop cycle (Vadez et al., 2013). This system allows for the measurement of highly relevant agronomic assessment of yield and yield components. At the same time, total plant water use (the sum of water use data across the entire life of the crop), dynamics of plant water use (water used during crops development), and transpiration efficiency [TE, the ratio between total biomass produced (pods and vegetative parts) and total water use] can be followed. Lysimetric assessment allows us to evaluate temporal variations in water requirements of individual genotypes (Vadez et al., 2011, 2013; Zaman-Allah et al., 2011; Vadez and Ratnakumar, 2016; Tharanya et al., 2018; Sivasakthi et al., 2019) and different crops like sorghum, pearl millet, finger millet, chickpea, and peanut. Using this facility, genetic variations for TE and grain yield in pearl millet were reported (Vadez et al., 2013; Tharanya et al., 2018). In addition, genotypic differences in water extraction pattern in critical crop growth stages were found using the lysimeter facility where drought-adapted genotypes were found to extract lesser quantity of water before flowering and to extract more water post-anthesis, which might confer adaptation to terminal drought (Vadez et al., 2013). Selection can be based on phenology, TE, grain yield, and pre- and post-flowering water utilization patterns. Screening for drought adaptation using pots has a distinct advantage to create fully controlled water stress and to record observations using complex and sensitive equipment. Several experiments assessed the transpiration response of pearl millet to atmospheric and soil drought under fully watered and water deficit conditions where drought-adapted genotypes showed a lower transpiration rate than drought-sensitive genotypes (Kholová et al., 2010a,b, 2016; Choudhary et al., 2019). Although phenotyping through pot culture is simple and cost-effective, it is very difficult to screen large populations with sufficient replication for traits like leaf area, as it involves a destructive method, and assessing transpiration could be laborious.

### Validation in Targeted Environment (Multilocation Trial, Managed Stress Environments)

Screening under field conditions is performed by evaluating the test material through multi-locational trials conducted in drought-prone regions (Yadav et al., 2012b) or by growing pearl millet during a rain-free season with adequate water supply but withholding irrigations to expose pearl millet to drought in the desired stage (Bidinger et al., 2000).

### Spatial Distribution of Crop Stress Maps in India Using Spectral Matching Techniques

**FIGURE 5:**
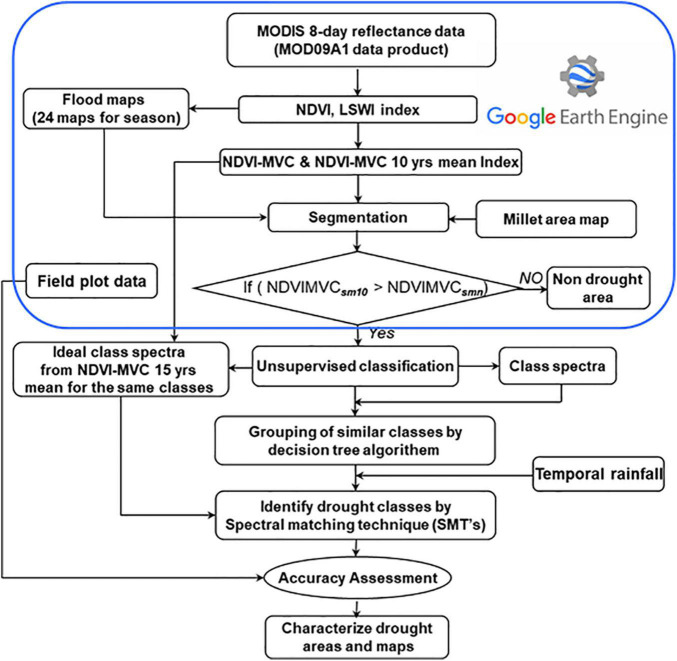
Flow diagram for drought characterization using spectral matching technique.

Assessing millet crop stress using Earth observation data in public domains, such as the Normalized Difference Vegetation Index (NDVI) product derived from the Moderate Resolution Imaging Spectroradiometer (MODIS), challenges multidisciplinary teams to understand different stresses in millet-growing areas. The process involves analyzing historical time series satellite imagery (Figure 5). Use of MODIS NDVI and LSWI products to map crop stress areas and changes in millet areas is due to rainfall. A methodology was developed to identify crop stress-affected areas using the MODIS-based NDVI and LSWI, and compare these crop areas with those during a normal year (Gumma et al., 2015). A long-term average of NDVI during the rainy (*Kharif*) season (June-October) is compared to a the current crop year. Rainy season NDVI and crop area changes are identified. Secondary data are used to support the crop stress analysis. Spectral matching techniques are utilized to categorize crop stress-affected cropland areas into three classes, severe, moderate, and mild, based on the intensity of damage assessed by field sampling. Spectral signatures are generated based on these ground survey samples (Gumma et al., 2019).

## Breeding Drought-Tolerant Pearl Millet

### Conventional Breeding

Although pearl millet is an inherently drought-tolerant species, breeding programs have targeted to further increase drought tolerance utilizing available genetic variations in its germplasm. Adaptive evolution concurrent with climate change in its center of diversity and area of production shaped the crop to successfully grow in areas of limited soil moisture (Serba and Yadav, 2016).

#### Trait Manipulation

Breeding programs have targeted mostly the mid-season and terminal drought stresses in pearl millet, assessed the importance of drought-adapted germplasm, and explored the usefulness of genetic introgression.

Tillering ability is the most important trait that has been strategically manipulated in mid-season drought stress breeding. There is a huge amount of variation in the tillering capacity of pearl millet and it has been reported to be a moderately heritable trait (Appa Rao et al., 1986; Rai et al., 1997; Yadav et al., 2017). Greater tillering provides elasticity to the growth and development of pearl millet and is part of its mechanism for adaptation to severe drought conditions. Several drought tolerance studies conducted in the Sahel have indicated that pearl millet is tolerant to water deficit until early grain filling, predominantly because the main shoot can be compensated by basal tillering (Winkel et al., 1997).

Earliness and short and rapid grain filling period are the two most important traits that have been manipulated for enhancing tolerance to terminal drought. Early flowering essentially determines grain productivity in water stress (Bidinger et al., 1987b; Fussell et al., 1991; Van Oosterom et al., 1995). Genetic variation for earliness is widely available in the germplasm (Rai et al., 1997; Yadav et al., 2017) and phenotype-based selection was accomplished (Rattunde et al., 1989). The frequently exploited basis of earliness is the Iniadi-type landraces collected from western Africa (Andrews and Kumar, 1996). Promising lines with the early flowering trait have been developed from *Iniadi* landraces and adopted in Indian and African agro-ecologies.

The proportion of panicle threshing denotes seed setting potential under low soil moisture conditions and integrating the effects of assimilation and translocation of photosynthates in drought environments is a measure of drought tolerance (Bidinger et al., 1987b). It exhibits a wide amount of variation in grain yield (Fussell et al., 1991; Bidinger and Mahalakshmi, 1993), is highly heritable (Yadav, 1994), and selection is effective (Bidinger and Mahalakshmi, 1993). Some mathematical models have also been used to recognize lines that are performing well under adverse conditions by comparing grain yields between stress and non-stress (optimum) conditions (Bidinger et al., 1987b; Yadav and Bhatnagar, 2001). Accordingly, multi-environmental data from a diverse range of growing conditions are used to identify drought-tolerant genotypes.

#### Role of Drought-Adapted Germplasm and Usefulness of Genetic Introgression

Successful growth of pearl millet in water stress is more of a reflection of its adaptation to drought stress than genetic yield potential (Bidinger et al., 1994). As adaptation is much less understood physiologically and genetically, it is more difficult to improve for adaptation than for yield potential. Pearl millet landraces that evolved in dry areas as a result of natural and man-made selection over thousands of years demonstrate better adaptability to water stress (Yadav et al., 2000; Yadav, 2010, 2014). Efforts were made to utilize these landraces in pearl millet conventional breeding approaches. Cycles of mass selection in genetically heterogeneous landraces are found to increase yield considerably (Bidinger et al., 1995; Yadav and Manga, 1999; Yadav and Bidinger, 2007) and have been revealed as a valuable germplasm source to breed drought-tolerant lines (Yadav, 2004) and develop inbred pollinator lines for hybrid breeding (Yadav et al., 2009, 2012a).

A huge amount of genetic variation in the global germplasm of pearl millet is available for traits that are constitutive and responsive to drought (Table 1). Murty et al. (1967) characterized 1,532 accessions and reported variation in days to flowering among Indian (52–77 days) and exotic (53–85 days) collections. In another study, days to flowering showed much wider variability (33–140 days) among a world collection of 16,968 pearl millet accessions (Harinarayana et al., 1988). IP 4021 (Bhilodi), collected from Gujarat, was the earliest to flower with 33 days to 50% flowering in rainy season and 34 days in post-rainy season. Furthermore, screening efforts identified two promising drought-tolerant lines among 509 accessions that were screened (Harinarayana et al., 1988). Landraces identified from Rajasthan, India exhibited wide variability for different characters. SR 15, SR 17, and SR 54 were three landraces that were identified for earliness. Three other unique landraces that showed earliness were “Chadi” from Rajasthan, “Bhilodi” from Gujarat, and “Pittaganti” from Eastern Ghats of India (Kumar and Appa Rao, 1987). Furthermore, germplasm accessions IP 4066, IP 9496, and IP 9426 were identified as promising early lines. In a detailed study conducted on a wide range of environmental conditions, 105 landraces were evaluated, a wide range in drought response was observed, and 15 landraces (IP 3243, IP 3228, IP 3424, IP 3296, IP 3362, IP 3180, IP 3272, IP 3303, IP 3252, IP 3258, IP 11141, IP 3318, IP 3123, IP 3363, and IP 3244) having a high degree of drought tolerance have been identified for use in developing drought tolerant cultivars (Yadav et al., 2003).

**TABLE 1:** Range of variation in pearl millet germplasm collection.

Character	Range	Mean
Time to 50% flowering-R (days)	33–159	72.7
Time to 50% flowering-PR (days)	32–138	71.7
Plant height (cm)-R	30–490	248.5
Plant height (cm)-PR	25–425	161
Total tillers (No.)	1–35	2.7
Productive tillers (No.)	1–19	2.1
Panicle exsertion (cm)	–45–29	3.5
Panicle length (cm)-R	5–135	28.9
Panicle length (cm)-PR	4–125	25.8
Panicle width (mm)-R	8–58	24
Panicle width (mm)-PR	8–61	22.8
1000-Seed weight (g)	1.5–21.3	8.5

*R, rainy season; PR, post-rainy season (adopted from Yadav et al., 2017).*

Differential response of landraces and elite genetic materials across contrasting drought and non-drought environments provided need of amalgamating drought tolerance of landraces and high-yielding potential of elite genetic material (Yadav, 2006, 2010; Yadav and Rai, 2011). Crosses between adapted landraces and elite genetic materials showed enhanced adaptation to drought and high productivity (Presterl and Weltzien, 2003; Yadav, 2008; Yadav and Rai, 2011; Patil et al., 2020). Selection of specific landraces and elite materials for hybridization is governed by combinations of phenotypic traits (like tillering, panicle size, seed size, height, and earliness), drought adaptation, and yield potential in two groups of materials (Patil et al., 2020).

The development of hybrids using strategic use of a diverse range of germplasm has been a top priority in pearl millet breeding, especially in India. However, it is generally argued that hybrids are more suitable for optimum environments than heterogeneous OPVs, as the latter has a population buffering effect to provide stable performance in unpredictable drought environments. A comprehensive study reported a yield advantage of hybrids ranging from 19 to 35% over OPVs (Yadav et al., 2012b). The current seed delivery system for pearl millet in India also favors hybrids, but this is not the case in Africa. Nevertheless, hybrids are more likely to play a key role than composites in enhancing pearl millet productivity in water stress environments.

### Genomics-Assisted Breeding

#### Development of Genomic Resources

During the past more than two decades, hundreds of thousands of molecular (DNA) markers have been developed and used in pearl millet genetic studies in different research stations around the world (Srivastava et al., 2020a). Different sorts of DNA-based markers like; RFLP, AFLP, RAPD, STSs, gSSRs EST-SSRs, DArTs, CISP, SSCP-SNP, and SNP markers have been devised and used to accelerate the pace of pearl millet breeding programs around the world (Liu et al., 1994; Devos et al., 1995, 2000; Allouis et al., 2001; Qi et al., 2004; Bertin et al., 2005; Senthilvel et al., 2008, 2010; Rajaram et al., 2010; Supriya et al., 2011; Sehgal et al., 2012, 2015; Tara Satyavathi et al., 2021). Several different molecular markers were used to assess genetic variability and population structure and detect genomic regions associated with agriculturally important traits through genome-wide association study (GWAS), genomic selection (GS), marker-aided breeding (MAB), and other genetic studies to speed up breeding programs in pearl millet (Table 2). DNA markers help determine the degree and quantum of genomic diversity in pearl millet germplasm to identify promising lines for hybridization and analyze population structure and QTLs linked with environmental stress tolerance (Vadez et al., 2012).

**TABLE 2:** Details of molecular markers developed for pearl millet related to genetic diversity, genome mapping, micronutrient, grain yield, fodder biomass, and stress estimation.

Sl. No.	Molecular marker	References
1.	Reported large-effect Fe and Zn content QTLs using DArT and SSR markers to construct a genetic linkage map with 317 RIL population developed from ICMS 8511-S1-17-2-1-1-B-P03 × AIMP 92901-S1-183-2-2-B-08 cross.	Kumar et al., 2018
2.	Pearl millet genome sequencing data was used to establish marker trait associations for genomic selection, to define heterotic pools, and to predict hybrid performance.	Varshney et al., 2017
4.	A set of 305 loci were used to construct a linkage map to map two QTLs for grain Fe content on LG3 and LG5, and two QTLs for grain Zn content on LG3 and LG7 using replicated samples of 106 pearl millet RILs (F_6_) derived from ICMB 841-P3 × 863B-P2 cross.	Kumar et al., 2016
5.	Genotyping data derived by 256 DArT and 70 SSR markers on 168 F_7_ recombinant inbred lines (RILs) from cross 81B-P6 × ICMP 451-P8 were used to construct a linkage map	Ambawat et al., 2016
6.	Identified 83,875 SNPs within five hundred pearl millet accessions, consist of 252 accessions and 248 Senegalese landraces, with genotyping by sequencing (GBS) of *Pst*I-*Msp*I reduced representation libraries.	Hu et al., 2015
7.	Thirty seven SSRs and CSIP markers have been developed, spanning 7 LGs evaluated in irrigated and drought stress conditions, 22 SNPs and 3 InDels for abiotic stresses	Sehgal et al., 2015
8.	ISSR-based SCAR marker have been devised for downy mildew (DM) resistance in pearl millet and associated to DM resistance LG with genetic linkage distance of 0.72 cM	Jogaiah et al., 2014
9.	75 SNPs and CISP were developed from EST sequences using parents of two mapping populations for 18 genes	Sehgal et al., 2012
10.	A array 574 polymorphic DArT markers was used to genotype a set of 24 diverse pearl millet inbred lines	Supriya et al., 2011
11.	A set 306 AFLP markers were developed and used in unravel genetic basis of pearl millet adaptation along an environmental gradient by a combination of genome scan and association mapping	Mariac et al., 2011
12.	Hundreds of polymorphic EST-derived SSRs were developed and deployed in mapping of RIL populations in pearl millet	Rajaram et al., 2010, 2013
13.	About 300 DArT markers have been used for polymorphic in different pearl millet RIL populations	Senthilvel et al., 2010
14.	A set of 30 random decamer oligonucleotides (RAPD) used in genetic diversity study	Govindaraj et al., 2009
15.	Cross-transferability of the 31 finger millet EST-SSRs were evaluated and found to be polymorphic in pearl millet	Arya et al., 2009
16.	A total of 306 AFLP and 27 SSR markers were identified and used in genotyping of 90 inbred lines in association studies in pearl millet	Saïdou et al., 2009
17.	Four EST-derived SSRs and 9 CISPs were used in linkage mapping using biparental mapping populations of pearl millet	Yadav et al., 2008
18.	A panel of 21 functionally informative EST-based SSRs and 6 gSSRs were developed in pearl millet	Senthilvel et al., 2008
19.	A set of 224 polymorphic AFLP markers have been developed and employed for genetic diversity assessment in south-western Niger	Allinne et al., 2008
20.	A panel of 19 EST-SSRs, among them 11 amplified and 4 were appeared polymorphism on agarose gels	Yadav et al., 2007
21.	A set of 16 EST-based polymorphic SSR markers were developed and used to assess genetic diversity of wild and cultivated pearl millet accessions of Niger	Mariac et al., 2006
22.	SSCP-SNP primes were developed through comparison of rice and pearl millet EST collections	Bertin et al., 2005
23.	Genetic maps developed in four different crosses were integrated to generate a consensus map of 353 RFLP and 65 SSR markers.	Qi et al., 2004
24.	Eighteen potential SSR markers were developed from genomic sequences in pearl millet	Budak et al., 2003
25.	RFLP probes were used to assess genetic diversity within and between 504 landraces of core collection using a subset comprising 10 accessions of Indian origin	Bhattacharjee et al., 2002
26.	A set of 25 SSR markers have been developed from 40 BAC pools, comprising a total of 384 clones	Qi et al., 2001
27.	A set of 42 (GT)n and 8 (CT)n microsatellites have been isolated from BAC clones pooled from a single 384-well microtiter plate in pearl millet	Allouis et al., 2001
28.	Genetic variability within and between pearl millet landrace estimated using variation at 163 amplified fragment length polymorphism marker (AFLP) loci	Busso et al., 2000
29.	AFLP markers were developed for the pearl millet using nuclear genomic sequences	Devos et al., 1995
30.	Study of a sample of varied pearl millet lines with 200 genomic DNA probes revealed pearl millet species to be highly polymorphic	Liu et al., 1994

In pearl millet, molecular mapping work started with the RFLP technique (Liu et al., 1992, 1994). One set of potential AFLP markers was developed and employed for genetic diversity assessment in south-western Niger (Allinne et al., 2008) and genomic regulation adaptation in new environments using the advantage of genome scan and association mapping (Saïdou et al., 2009; Mariac et al., 2011). Mariac et al. (2006) developed novel microsatellite markers in pearl millet using public expressed sequence tags (ESTs) available on GenBank.

Subsequently, 21 EST-derived SSRs and six genome-based SSRs were devised from 3,520 EST sequences and employed in genetic mapping in pearl millet (Senthilvel et al., 2008). The design of 277 DArT markers from 6,900 DArT clones utilized *Pst*I/*Ban*II complexity reduction using RIL population (Senthilvel et al., 2010). In another study, a set of robust DArT-based SNPs were detected from several hundred DArT clones generated from different accessions through the *Pst*I/*Ban*II complexity reduction protocol in millet inbred lines (Supriya et al., 2011). Seven LGs covering 1,749 cM with inter marker distance of 5.73 cM were determined by the mapping of DArT and SSR markers; 2 co-localized QTLs associated with grain Fe and Zn content were detected in LG 3 in pearl millet species (Kumar et al., 2016).

Genomic resources have been exploited to generate CISP and SNP markers employed in the discovery of candidate genes pertaining to drought-tolerant QTLs in different accessions in pearl millet (Sehgal et al., 2012). Under another breakthrough, thousands of SNPs were developed through genotyping-by-sequencing (GBS) protocols in global germplasm collections and landraces in pearl millet lines (Hu et al., 2015; Serba et al., 2019; Kanfany et al., 2020). Besides, ISSR-derived SCAR markers have been developed for the analysis of genetic diversity among parental lines used to develop mapping populations for studies on downy mildew (DM) resistance in pearl millet. A distinct locus has been detected in ICMR 01004 line, which was confirmed with PCR technique. The detected SCAR marker was ultimately endorsed in different accessions collected from wide-geographical locations, and the findings indicate its linkage to DM resistance in pearl millet (Jogaiah et al., 2014). A linkage map was constructed by integrating DArT and SSR markers, which were employed in the detection of rust resistance QTLs in LG1 in pearl millet (Ambawat et al., 2016). With the advancement of next-generation sequencing (NGS) technologies, rapid decoding of the crop has enabled the availability of a huge number of genome-wide single nucleotide polymorphism (SNP) markers for trait mapping, trait selection, and trait improvement gains in pearl millet (Table 3). These markers are proved to be essential genomic tools for various genetic studies on pearl millet for crop improvement by molecular breeding (Srivastava et al., 2020b).

**TABLE 3:** Key sequence-based markers and their application in various genomic studies on pearl millet.

Approach	Trait mapped/studied	Key features	References
GBS-based SNPs	Early- and late-flowering	A panel 21,663 single nucleotide polymorphisms (SNPs) markers with more than 5% of minor allele frequencies were discovered	Diack et al., 2020
	Genetic diversity, population structure and linkage disequilibrium	By mapping the GBS reads to the reference genome sequence, 82,112 genome-wide SNPs and screened on a total of 398 accessions to assess genetic diversity, population structure, and linkage disequilibrium.	Serba et al., 2019
	Genetic diversity	Genetic diversity of 130 forage-type hybrid parents of pearl millet was investigated using GBS-derived 7870 SNPs	Ponnaiah et al., 2019
	Genomic diversity	Genomic diversity assessed in 309 pearl millet inbred lines derived from landraces and improved varieties using 54,770 high quality SNP markers	Kanfany et al., 2020
	Cytoplasmic-genic male-sterility	QTL explained only 14.5% and 9.9% of the phenotypic variance of pollen production and selfed seed set	Pucher et al., 2018
	Drought	A total of 392,493 SNPs identified and used for QTL analysis	Debieu et al., 2018
	Diversity	Produced several tens of thousands of single nucleotide variants, but differed in the way the variants were identified	Berthouly-Salazar et al., 2016
	Leaf Spot Resistance	16,650 single-nucleotide polymorphisms and 333,567 sequence tags spread across all seven chromosomes	Punnuri et al., 2016
	Striga resistance	Identification of genomic regions associated with Striga resistance and other important agronomic traits	Moumouni et al., 2015
	Population genomics	Identified 83,875 SNPs in 500 accessions, (252 global accessions and 248 Senegalese landraces) using GBS of *Pst*I-*Msp*I reduced representation libraries and used to characterize genomic diversity and population structure.	Hu et al., 2015
	Heterotic groups	343 hybrid parental [maintainer (B-) and restorer (R-)] lines genotyped with 88 polymorphic SSR markers, clustered in 10B- and 11R- groups 0.9 million SNPs clustered into 12 R- and 7 B-line groups	Ramya et al., 2018; Gupta et al., 2020
RAD-GBS	Heat and drought	Identify 6920 genes and 6484 genes differentially expressed under heat stress and drought stress	Sun et al., 2020
	Genomic prediction and genomic selection	Three GS models implemented and compared using grain yield and dense molecular marker information of pearl millet obtained from two different genotyping platforms (C [conventional GBS RAD-seq] and [tunable GBS tGBS]).	Jarquin et al., 2020
	Genomic selection	Evaluated two genotyping techniques and four genomic selection schemes in pearl millet. Sequencing data were generated for RAD-seq and tGBS.	Liang et al., 2018
RNAseq	Terminal drought tolerance	Underlined flavanoid pathway, lignin biosynthesis, phenyl propanoid pathway, pigment biosynthesis, and other secondary metabolite pathways in drought stress at flowering stage than at the vegetative stage.	Shivhare et al., 2020
	Drought tolerance	A total of 6799 and 1253 differentially expressed genes were found in ICMB 843 and ICMB 863 ilnes, respectively under drought stress	Dudhate et al., 2018
	Drought tolerance	A total of 19983 differentially expressed genes, 7595 transcription factors, gene regulatory network having 45 hub genes controlling drought response	Jaiswal et al., 2018

#### Genetic Linkage Maps

The first milestone was achieved with the development of the shortest map (303 cM) with 181 RFLP markers using an F_2_ pearl millet mapping population (Liu et al., 1994). Later, an integrated consensus linkage map of all 7 genetic maps was developed using different crosses with RFLP and SSRs (Qi et al., 2004). In an ICMB 841 × 863 B cross, 91 loci were incorporated using F_2_ progenies with a 617.4-cM map length (Yadav et al., 2004). A total of 21 informative SSR loci were mapped using previously developed two bi-parental populations derived from crossing an ICMB 841-P3 line with 863B-P2 and 81B-P8 × IPC 804 lines showed that all functional SSRs mapped on distant regions of LGs cover the preceding gaps (Senthilvel et al., 2008). To construct a linkage map with uniform marker distribution with improved coverage of gaps in existing genetic maps, a linkage map was developed using 196 different types of molecular markers with a mean inter-marker distance of 9.2 cM (Pedraza-Garcia et al., 2010). A linkage map was developed with a set of 258 DArT and 63 polymorphic SSR genotyping data using a 140 F_7_ recombinant inbred line (RIL) population derived from the H77/833-2 × PRLT 2/89-33 cross to construct a dense linkage map by integrating DArT and SSRs (Supriya et al., 2011). This relatively improved linkage map consisted of 321 loci spanning 1,148 cM with a mean inter-marker distance of 3.7 cM, and the length of individual LGs varied between 78 (LG 3) and 370 cM (LG 2) (Supriya et al., 2011). A longer skeleton linkage map of 1,019 cM length for F_2_-bred F_3_ progenies spanning 44 uniformly distributed loci with an inter-marker distance of 14 cM (LG1) to 38 cM (LG6) and an overall average distance of 23 cM has been reported by Vengadessan et al. (2013).

Genetic linkage maps were constructed with 99 in-house designed novel EST-SSRs and 17 previously mapped SSRs, 53 gSSRs, and 2 informative STS markers (Rajaram et al., 2013). A consensus map spanning 1,74 loci covering 899 cM genetic distance was constructed by integrating different linkage maps with the MergeMap tool, which revealed an evolutionarily conserved locus order for almost each LG (Rajaram et al., 2013). Then, a genetic linkage map was constructed comprising SNPs with F_2_ population of a wild × domesticated cross with improved coverage and improved consistency of loci compared to formerly released maps. The linkage map was developed with a revised GBS protocol involving two (*Pst*I and *Msp*I) restriction enzymes and PCR amplification with primers comprising 3 discerning nucleotides to generate thousands of SNPs for open access (Moumouni et al., 2015). Linkage maps were developed with 305 markers (96 codominant SSRs and 208 dominant DArTs), which detected seven LGs spanning 1,749 cM with a mean distance of 5.73 cM among loci. A panel of 106 RILs (F_6_) derived from a cross between ICMB 841-P3 and 863B-P2 lines was used in the study of Kumar et al. (2016). Additionally, a genetic linkage map was also constructed with DArT and SSR markers using 317 RIL progenies derived from ICMS 8511-S1-17-2-1-1-B-P03 × AIMP 92901-S1-183-2-2-B-08, a bi-parental cross of ICMS 8511-S1-17-2-1-1-B-P03, and AIMP 92901-S1-183-2-2-B-08 lines. The base map (LGs) of 196 loci was 964.2 cM in length (Kumar et al., 2018). The calling of a huge number of SNPs and the availability of saturated genetic maps may accelerate the pace of progress in linkage studies using cross populations considerably and make easy genetic studies on collections of diverse lines.

#### Genomic Regions/QTLs

Thousands of informative DNA markers have been deployed to dissect QTLs for investigation of the molecular and biochemical bases of tolerance to abiotic stress in millets, and to devise an efficient approach for crop improvement for yields under drought stresses. Subsequently, dissection of QTLs pertaining to drought tolerance (DT-QTLs) and high grain yield was identified in independent studies using different pearl millet mapping populations (Yadav et al., 2002, 2004; Bidinger et al., 2007). The milestone breakthrough in detection of major DT-QTL in LG2 related to grain yield explaining 32% phenotypic variance was carried out (Yadav et al., 2002) using bi-parental populations of individuals of different crosses. Afterward, a minor QTL in LG5 linked with drought tolerance and explaining 14.8% phenotypic variance was also detected (Yadav et al., 2004). The putative drought-tolerant QTL in LG2 was evaluated using near-isogenic lines derived from H 77/833-2 into which a DT-QTL has been introgressed from PRLT 2/89-33 (Serraj et al., 2005). In the same direction, three major QTLs (positioned in LG2, LG3, and LG4) pertaining to grain yield with limited QTL × environment interactions were analyzed as key components of MAB for improved grain yield under variable post-flowering water stresses in pearl millet (Serraj et al., 2005; Bidinger et al., 2007). Major QTLs mapped on LG2 and LG3 accounted for a wide (13-25%) range of phenotypic variations for grain yield traits under drought stress conditions. At the same time, minor QTLs were also co-mapped for harvest index under drought stress, and QTLs for both grain number and individual grain mass in water deficits were identified (Bidinger et al., 2007).

These QTLs mapping findings have been validated in linkage mapping investigations that mapped QTLs linked to high grain yield and its related traits under terminal drought stresses in pearl millet (Yadav et al., 2011). In this study, one major QTL associated with grain yield and drought tolerance of grain yield under water stress conditions was detected in LG2, which explains about 32% of phenotypic variance in test cross progenies (Yadav et al., 2011). The effect of this major QTL that explained 32% of the variance under drought stress was confirmed in different marker-aided backcross programs, where a 30% increment for grain yield general combining ability (GCA) anticipated of DT-QTL in water deficits was recovered in QTL introgression lines (Yadav et al., 2011). Millet species have evolved with an inherited potential for climate resilience to biotic and abiotic stresses, and they may serve as an important source of key genes underlying stress tolerance, which have to be further investigated and characterized (Jangra et al., 2019). The close taxonomic relationship of millets with other staple cereal crop species may allow for introgression of untapped alleles, key genes, genomic regions, or QTLs detected in millets for improved agro-economic traits into other cereals to ensure sustainable food and nutritional security under climate changes and increasing global warming. Details of the genomic regions/QTLs associated with drought stress and other component agronomic traits in pearl millet are provided in Table 4. To dissect the components of drought-tolerant QTLs, 75 SNPs and CISP markers have been designed using an open-access EST database created for some elite pearl millet lines (Sehgal et al., 2012). Some key regulatory genes encoding photosystem I (PSI) reaction center component proteins were identified from DT-QTL regions mapped on LG2 (Sehgal et al., 2012); these candidate genes are supposed to contribute to QTL regions associated with yield and flowering traits under water stress in pearl millet.

**TABLE 4:** Details of genomic regions/quantitative trait loci (QTLs) associated with drought stress and other component agronomic traits related to drought in pearl millet.

Sl. no.	Genomic regions/QTLs	Controlled traits	References
1.	Drought tolerance (DT)-QTLs introgression	DT-QTLs introgressed into hybrid HHB 226 from 863 B, the male parent HBL 11	Jangra et al., 2019
2.	Five QTLs identified using LD/association mapping	Biomass production in early drought stress conditions and stay-green trait	Debieu et al., 2018
3.	F7 recombinant inbred lines were used to identify 4 QTLs and allelic interactions for traits affecting plant water use	QTL detected linked to high transpiration rate under high vapor pressure deficit, alleles from drought-sensitive parent ICMB 841. A major QTL was mapped on LG6	Aparna et al., 2015
4.	Re-assessed DT-QTL on LG 2 for control of ion uptake under salinity stress during post-flowering stages	associated with reduced salt accumulation and compartmentalization conferred by alleles at DT-QTLs on LG 2	Sharma et al., 2014
5.	A total of SNPs were added to the positions of major DT-QTLs on LG 2 were to the existing function map	identified 18 underlying candidate genes with DT-QTLs	Sehgal et al., 2012
6.	Alleles co-mapped to terminal drought tolerance QTL with small genetic interval	alleles controlling transpiration rates under fully irrigated conditions	Kholová et al., 2012
7.	One major DT-QTL on LG 2 salt uptake	associated with reduced salt uptake and exerts favorable effects on growth and yield by limiting Na^+^ accumulation under salt stress	Sharma et al., 2011
8.	One major QTL on LG 2	grain yield and drought tolerance of grain yield in drought stress environments	Yadav et al., 2011
9.	QTL responsible for terminal drought tolerance	associated with lower transpiration rate and control of leaf water losses in under well-watered conditions	Kholová et al., 2010a
	Three major QTLs on LG 2, LG 3, and LG 4	grain yield under variable post-flowering water conditions	Bidinger et al., 2007
10.	First assessment of a putative QTL LG 2 of pearl millet	drought tolerance trait	Bidinger et al., 2005
11.	Putative DT-QTL on LG 2	evaluated in near-isogenic versions of H 77/833-2 into which drought tolerant QTL has been introgressed from PRLT 2/89-33	Serraj et al., 2005
12.	One minor QTL on LG 5	post-flowering drought tolerance	Yadav et al., 2004
13.	One major QTL on LG 2	grain and stover yield in pearl millet under terminal drought stress conditions	Yadav et al., 2002

The key function of a major DT-QTL was tested, and one major terminal DT-QTL was found to be controlling minimum salt uptake and better adaptation under salt-induced oxidative conditions in pearl millet. An illustration of restrained salt acquisition in a drought-resilient accession was found to invariably be linked with improved plant growth with grain yield in salt stresses. There was evidence that the DT-QTL conferred positive impacts on luxurious crop development and yield characters under saline conditions by reducing the accumulation of sodium ions in aerial parts of plants (Sharma et al., 2011). The major QTL linked with improved drought tolerance in the post-flowering and grain-filling stages has also been found to be associated with limited salt uptake and transport in pearl millet (Sharma et al., 2014). Reduced levels of toxic (Na^+^) ionic accumulation and compartmentalization have been recorded in leaves, while access accumulation of toxic ion Na + was diminished in plant roots of pearl millet. It may be concluded that ionic uptake is controlled by genes pertaining to the DT-QTL in LG2 (Sharma et al., 2014). The QTL associated with low transpiration rates that contributes to water stress tolerance by lodging soil water contents to be used in the grain filling stage by limiting moisture loss in the vegetative phase has been co-mapped with the terminal DT-QTL (Kholová et al., 2012). Low rate of transpiration is maintained by physiological and morphological interactions (Kholová et al., 2012). To unravel the physiological mechanisms behind DT-QTL, fine mapping population segregating for DT-QTL was used to co-map the QTL for transpiration rate (TR) and established that by minimizing water loss during vegetative stage, DT-QTL conserves soil moisture and, thus, contributes to terminal drought tolerance (Kholová and Vadez, 2013). Furthermore, these DT-QTLs associated with terminal drought tolerance have been introgressed into elite pearl millet lines to improve terminal drought tolerance (Jangra et al., 2019). Marker-aided foreground selection has been performed with robust SSR markers mapped on LG2 and LG5 to select plants harboring alleles that render resistance to bi-parental progenies (BC4F2) along with rigorous phenotypic selection to recover the genome of the recurrent parent in pearl millet (Jangra et al., 2019).

#### Transcriptome Assemblies

Transcriptomic investigation with next-generation sequencing (NGS) technologies has emerged as an efficient proficient approach for analyzing gene expression profiling in plant species. However, RNA sequencing (RNA-seq) utilizing the NGS technique can detect differentially expressed genes (DEGs) owing to their dynamic range of expression levels (Jongeneel et al., 2001). A *de novo* assembly based transcriptome analysis under drought stress was published and reported several key genes, transcription factors, gene regulatory networks that regulate water stresses in pearl millet (Jaiswal et al., 2018). Some informative SNPs were identified controlling nucleic acid and amino acid metabolic pathways under environmental stresses. Since the pearl millet whole-genome sequence has been published (Varshney et al., 2017), attempts were made to unveil the genetic basis of tolerance to water stresses in pearl millet through the RNA-seq approach (Dudhate et al., 2018). More recently, an important study on RNA-seq analysis was performed to assess the comparative transcriptomics in the vegetative and reproductive stages to discover key genes involved in drought response in a pearl millet drought-tolerant (PRLT2/89-33) line (Shivhare et al., 2020). Several underlying genes, pathways involved in lignin, flavonoids, pigments, and other important secondary metabolites for biosynthesis were enriched and activated in pearl millet (Shivhare et al., 2020).

#### Marker-Assisted Forward Breeding

Genomics provides opportunities to develop DNA-based tools to mitigate the problems of ever-increasing food and fodder yield, nutritional security, and sustainable crop production by modern breeding approaches. The development and deployment of DNA markers for plant genetic advancements through marker-assisted selection (MAS) have been established in bi-parental breeding. Advances in plant genomics offer high-density sequence-based molecular tools exploiting NGS technology for genetic evaluation of breeding populations, and are important to accelerate the pace of pearl millet breeding programs (Varshney et al., 2017). A genomics-driven breeding scheme for breeding of climate-resilient varieties (Varshney et al., 2005) has been initiated by dissecting abiotic stress that will likely influence grain yield and productivity in prevailing climate change due to global warming. Information derived from multi-environment trials offers an opportunity for studying the impacts of abiotic stress on crop plants and populations under investigation. Crop scientists are involved in the exploration of morphological and physiological traits in plants that could improve crop adaptation to this looming climate change. In the present context, plant physiology facilitates the description of ideotypes to be practiced for improving salient adaptation under such harsh climate scenarios. Additionally, the application of geographic and passport information permits the selection of lines thriving well in abiotic stress-prone locations, while existing techniques such as genetic profiling, data analysis, and mapping of agriculturally important genes and genomic regions (QTLs) help in identifying potential lines for next-level screening for certain abiotic stresses (Kole et al., 2015). Moreover, accurate phenotyping for morphological traits and proper biometric evaluation facilitate the identification of certain responses of a panel of accessions in the prevailing growth stage affected by variable weather conditions. Such type of inputs has further been utilized in genomics-assisted breeding (GAB) techniques including genome-wide selection of promising accessions for further deployment in breeding and variety development (Srivastava et al., 2020a). Genetic mapping and QTL dissection using bi-parental and association mapping (AM) populations have facilitated the analysis of genomic regulation of expression agronomic traits. It also precisely permits marker-assisted selection (MAS), linkage mapping (QTLs), and GWAS and GS of promising lines to be developed in the perspective of breeding strategies (Kulwal et al., 2012; Kumar et al., 2018; Singh et al., 2020).

Until recently, the use of GWAS and GS has been impeded because of unavailability of high-density sequence-based DNA markers (Srivastava et al., 2020a). Invention and advancement in genomic technologies with NGS protocols have paved the way for rapid development of genome-wide molecular markers including SNPs and insertion-deletions (InDels) in pearl millet and other orphan plant species (Varshney et al., 2017). Identification of candidate genes and untapped alleles for target agronomic traits has been accomplished through GBS–based genetic approaches (Debieu et al., 2018). In the same way, GWAS has been conducted to detect genomic regions controlling agronomically useful traits and determine the statistical association between genetic polymorphisms and trait variations in area-wide germplasm collection subjected to genotypic and phenotypic characterizations. NGS in association with GWAS enhances high-resolution QTL/gene/s mapping (Varshney et al., 2017; Debieu et al., 2018; Serba et al., 2019). NGS-based technologies with cost-efficient genotyping assays, determination of genome-wide genetic variations, fine linkage mapping, and discovery of candidate genes for drought tolerance and natural allelic variants for QTLs controlling key agronomic traits have gained increased attention in routine plant breeding programs. For instance, three major QTLs linked to grain yield with low QTL and environmental (QTL × E) interactions were detected across different post-flowering water stresses in pearl millet (Bidinger et al., 2007). One of the major QTLs explained ∼32% of phenotypic variation in grain yield under water deficit conditions. The impacts of these dominant QTLs have been validated in marker-assisted back cross (MABC) programs wherein 30% enhancement of the general combining ability for grain yield was anticipated from this QTL under terminal drought stress. It was recovered in introgression lines using informative data generated with markers flanking the QTL (Yadav et al., 2011). This QTL has been fine-mapped using the LG2 QTL NIL-derived F2 mapping population with ddRAD SNPs (Srivastava et al., 2017). Out of 52,028 SNPs that were identified among NILs, a total of 10 SNPs were anchored to the QTL interval and are being used in forward breeding programs using the HTPG platform.

#### Marker-Assisted Backcrossing

Many potential marker-assisted backcrossing (MABC) methods are devised, which have been employed in QTL introgression from a donor to an elite recurrent parent, while pearl millet lines have been used to develop base mapping populations. ICRISAT (Patancheru, India), with the collaboration of United Kingdom-based scientists supported by the Plant Sciences Programme, Department for International Development (DFID), has made substantial efforts to develop and use genomic tools for increasing grain yield, yield stability, and productivity of hybrid cultivars in pearl millet (Hash et al., 1999). They described the use of MABC methods in pearl millet genetic improvement and attempted to enhance the drought tolerance of an elite inbred pollinator (H 77/833-2) line using a donor PRLT 2/89-33 line and the elite inbred seed parent maintainer genotype ICMB 841 using the 863B line as a donor. MABC methods have played a significant role in the considerable boost of grain yields, production, and quality in pearl millet, which has been achieved in India over the past many years. Development of a marker flanking QTLs governing yield and related traits under limited soil water contents has been a major focus of such type of research activities in the last several decades (Yadav, 2010, 2014). Several validated QTLs have been introgressed into elite hybrid parental lines (A-/ B-, R-), resulting in improved version of hybrids or essentially derived varieties (EDVs). Over two million people enjoy improved food security because of an output from these collaborative research programs using genomic resources in the breeding of genetically advanced pearl millet lines with high resilience to downy mildew. Efforts involving ICRISAT, John Innes Centre, Norwich; Institute of Grassland and Environmental Research, Aberystwyth University, University of Wales, Bangor, United Kingdom, and Chaudhary Charan Singh Haryana Agricultural University (CCSHAU), Hisar, India have detected QTLs associated with DM resistance in the pearl millet H 77/833-2 line, and 2 more QTLs were introgressed using MABC to design a male parental line with improved DM-resistance. The popular hybrid HHB 67 Improved was bred with a cross between improved DM resistant seed parent 843-22A and improved restorer parent H 77/833-2-202 lines. HHB 67 Improved is currently grown in more than 10% of the entire crop area every year in India and is one of the most impactful research outputs in crop plants globally. Recently, efforts were made to further improve the HHB 67 Improved hybrid, as it has started to show signs of breakdown to DM-resistance again. Several downy mildew resistance double QTL introgression lines were generated in the genetic background of restorer parent H 77/833-2-202. These improved introgression lines were used to produce HHB 67 Improved-like test-cross hybrids with enhanced levels of downy mildew resistance (Srivastava et al., 2015, 2020b). These second-cycle improved versions are being tested in EDV trials conducted by AICRP-PM. The MAS has been extensively employed in pearl millet to breed advanced versions of the previous lines (Sharma, 2001; Yadav et al., 2005; Hash et al., 2006; Sehgal et al., 2015). The validated LG2 terminal drought-tolerant QTL (Kholová et al., 2010a,b; Bidinger et al., 2007) from drought tolerant parent PRLT 2/8933 has been moved to H 77/8332 using MABC.

#### Genomic Selection

Improvement of genetic gains in terms of grain yield in harsh environments needs knowledge-based selection approaches that use phenotypic and genotypic data. Such type of marker-aided selection has been regarded as genomic selection (Meuwissen et al., 2001). The efficacy of genomic selection in augmenting the degree of genetic gain in crops and evaluation of the implementation of genomic models are limited to other millet crop species (Srivastava et al., 2020a). A first GS study on pearl millet was carried out by Varshney et al. (2017) at ICRISAT, Patancheru to predict grain yield for test crosses and then to predict the performance of single cross hybrids derived from a cytoplasmic-genetic male sterility (CMS) system in individual trials (Liang et al., 2018). High-throughput genotyping methods and genomic sequencing techniques have enabled the generation of rapid and cost-efficient genotypic assays, which facilitate the use of genomic prediction (GP) and genomic selection (GS) in plant and animal breeding. GS exploits DNA marker data to determine genomics-estimated breeding values (GEBVs) for a complex trait, and final selection is based directly on GEBVs exclusive of further field-based phenotypic characterization (Crossa et al., 2017). The key principle is that the genotypic data generated from several markers are uniformly distributed in plant genomes having the potential to unveil genomic polymorphism, and evaluate breeding values without prior information about where the selected alleles are located (Crossa et al., 2017).

Genomic selection (GS) in crop species is advantageous owing to its potential to predict selection decisions even in the off-season, hence enhancing significant genetic gain (Heffner et al., 2010). A simulation analysis was accomplished on maize and established the advantage of GS in place of the conventional marker-assisted selection approach (Crossa et al., 2013). Since then, several studies have exploited the benefits of GS in several crops including maize (Shikha et al., 2017), wheat (Norman et al., 2017), barley (Nielsen et al., 2016), sorghum (Fernandes et al., 2018), and pearl millet (Liang et al., 2018). Plant breeding studies have been reported on assessing the prediction ability of GS in various commercially useful crops, using different genotyping platforms and densities, and applying different parametric and non-parametric genomic prediction models to different agronomically important traits. Recently generated resequencing data have been used to perform GS to predict grain yield for test crosses in pearl millet. Different situations of prediction were explored in GS, for instance, the performance of grain yield within and across environments. High prediction accuracy has been recorded in terms of Pearson correlation coefficient among the predicted and observed values, optimized using the square root of the heritability for performance in diverse environments (Varshney et al., 2017).

Genome-wide molecular markers are potential tools for QTL discovery and deployment (Mohan et al., 1997; Bevan and Uauy, 2013). Moreover, high-density DNA markers are useful in the assessment of genetic variations and population structures of plant germplasm collections for systematic utilization in the breeding of elite varieties and genetic resource conservation and management. With the current progress made in NGS technologies and cost-efficient genotyping platforms, determination of genome-wide genetic variability, fine QTL mapping, and discovery of candidate genes for drought tolerance and natural allelic variants for QTLs controlling key agronomic traits have gained increased attention in routine plant breeding programs.

## Future Thrust

More than 70% of the pearl millet area would continue to be rainfed where moisture availability is seldom adequate, and the crop will always be prone to facing intermittent drought in the critical growth stage. Increasing climate variability in pearl millet production ecology and the burgeoning population in south Asia and sub-Saharan Africa imply a greater competition for water use in staple crop production besides the industry and domestic sectors. Current defined production ecologies are either vague or quite old, with high variations in agro-climatic conditions. Pearl millet target production environments require rigorous quantification of dynamic TPE with high resolution advanced MET database and crop coefficients to accelerate breeding programs. Enhancing crop productivity with less amount of water, the so-called concept of “more crop per drop,” is becoming a global necessity. Advancements in genomic tools and their effective integration improve the prospects of drought tolerance pipelines through target traits introgression and rapid recycling using speed breeding platform of adapted pearl millet germplasm/parents in conventional breeding. The availability of germplasm collections is relatively meager for diversifying the cultivar base in target regions. Therefore, the inclusion of West Africa and western India warrants more drought-adapted breeding populations. Adding new germplasm collections to drought-tolerant crossing programs to broaden the genetic base ensures the least disturbing maturity period (70–75 days) that is preferred by farmers. There existed a need of putting great thrust on the development of hybrid parents for drought-affected areas as hybrids have a distinct yield advantage of 20–30% over OPVs. ICRISAT exclusive drought-tolerant product profiles established in 2020 will provide the opportunity to build resources to the mainstream of validated traits, linked markers, and genomic selections with high-throughput screening facilities in target environments or platforms at the breeding center that mimic target environments.

Further success will depend on the development of repeatable, cost-efficient, high-throughput phenotyping facilities that reliably characterize genetic variation for drought tolerance and its contributing traits. Currently, hyperspectral remote sensing UAV technologies are an alternative to the manual collection of crop data, offering information on traits and factors influencing crop development and productivity with relatively shorter time and lower cost. Use of ultra-high spatial resolution hyperspectral remote sensing technology aided by machine learning and artificial intelligence (AI) have the potential to revolutionize the drought tolerance breeding process. These will aid in faster selection process and make more inclusive many traits that are complex or time consuming in assessment, like component traits for adaptation. Several new techniques for genomics offer a great opportunity to accelerate the cultivar development process. NGS-based genomic technologies have the potential to revolutionize the way crop breeding for drought tolerance is being attempted. Rapid trait mapping, validation, and deployment may be possible by combining advances made in our understanding of the underlying physiological factors and their interaction with environmental indices, and precision phenotyping. Marker-assisted deciphering of heterotic gene pools and development and diversification of the genetic base of seed and pollen parents will be critical in the development of heterotic and resilient drought-adapted cultivars. The heterotic group-based breeding strategy may bring viable breeding pipelines in the next few years using appropriate elite germplasm in crossing, rapid generation (RapidGen) advancement, and consortium-based drought trial network testing to stage-gate approaches. All such integrated programs with network trailing established and led by ICRISAT in association with NARS will accelerate the drought-tolerant product pipelines in India. Whole-genome resequencing-based selection approaches may help reduce the complexity associated with drought and its underlying component traits, and may provide useful insights into favorable haplotypes associated with drought adaptation. These can help us move toward breeding strategies based on haplotype selection leading to an enhanced rate of genetic gains in drought agro-ecologies.

## Author Contributions

RKS, RV, and OY planned and coordinated the study. RKS, OY, MaG, SG, DS, JK, SK, TM, SC, CS, RBS, SB, RG, and RV contributed to this work and drafted the manuscript. All authors contributed to the article and approved the submitted version.

## Conflict of Interest

The authors declare that the research was conducted in the absence of any commercial or financial relationships that could be construed as a potential conflict of interest.

## Publisher’s Note

All claims expressed in this article are solely those of the authors and do not necessarily represent those of their affiliated organizations, or those of the publisher, the editors and the reviewers. Any product that may be evaluated in this article, or claim that may be made by its manufacturer, is not guaranteed or endorsed by the publisher.

## Footnotes

1
http://gems.icrisat.org/leasyscan/

2
www.phenospex.com

3
http://gems.icrisat.org/lysimetric-facility/
